# Pediatric Integrative Medicine Approaches to Attention Deficit Hyperactivity Disorder (ADHD)

**DOI:** 10.3390/children1020186

**Published:** 2014-08-27

**Authors:** Anna Esparham, Randall G. Evans, Leigh E. Wagner, Jeanne A. Drisko

**Affiliations:** Program in Integrative Medicine, University of Kansas Medical Center, Kansas City, KS, 66160, USA; E-Mails: revans5@kumc.edu (R.E.); lwagner@kumc.edu (L.W.); jdrisko@kumc.edu (J.D.)

**Keywords:** pediatric, integrative, medicine, approach, ADHD, nutrient, diet, microbiome, environment, neurofeedback

## Abstract

Attention deficit hyperactivity disorder (ADHD) is the most common neuropsychiatric disorder in children and is increasing in prevalence. There has also been a related increase in prescribing stimulant medication despite some controversy whether ADHD medication makes a lasting difference in school performance or achievement. Families who are apprehensive about side effects and with concerns for efficacy of medication pursue integrative medicine as an alternative or adjunct to pharmacologic and cognitive behavioral treatment approaches. Integrative medicine incorporates evidence-based medicine, both conventional and complementary and alternative therapies, to deliver personalized care to the patient, emphasizing diet, nutrients, gut health, and environmental influences as a means to decrease symptoms associated with chronic disorders. Pediatric integrative medicine practitioners are increasing in number throughout the United States because of improvement in patient health outcomes. However, limited funding and poor research design interfere with generalizable treatment approaches utilizing integrative medicine. The use of research designs originally intended for drugs and procedures are not suitable for many integrative medicine approaches. This article serves to highlight integrative medicine approaches in use today for children with ADHD, including dietary therapies, nutritional supplements, environmental hygiene, and neurofeedback.

## 1. Introduction

Attention deficit hyperactivity disorder (ADHD) is characterized as a psychiatric condition of heightened impulsivity, inattention, and hyperactivity [1]. ADHD is the most common neurodevelopment disorder in children, affecting approximately 11% of children between 4 and 17 years of age in the United States. Increased public awareness and escalation of inciting risk factors has been accompanied by an increased rate of ADHD diagnosis, along with a rise in medication use [2]. The definitive etiopathogenesis of ADHD remains elusive due to its complex, multifactorial nature. ADHD has strong genetic and environmental influences [3]. However, the increased prevalence of ADHD is likely due to genetics but rather these environmental factors. Nutritional status, oxidative stress, neurotransmitter and endocrine dysregulation, neurological abnormalities in fronto-striatal and basal ganglia network, history of physical and emotional trauma, and environmental toxicity have all been implicated in ADHD [4,5,6,7,8,9]. In order to identify the most appropriate treatment, healthcare providers should identify these risk factors with a thorough evaluation of the child with ADHD.

It is crucial that children with ADHD are treated appropriately as they can develop significant psychosocial, educational, and neuropsychological impairment [10]. They are also at risk for not achieving their highest potential in education and employment as adults [11]. Behavioral therapy and medications are central to the management of ADHD, resulting in greater improvements in academic performance, reduction of behavioral problems, and higher parental satisfaction [12,13]. Limited access to mental health professionals interferes with the utility of psychosocial therapy. Only one-third of youths treated for ADHD received both psychosocial therapy and medications, and less than 10% received psychosocial interventions alone [14]. Children with ADHD also exhibit several comorbid disorders, resulting in decreased response to treatment.

Medication for ADHD is associated with its own set of problems. Twenty to 35% of patients with ADHD do not respond to medication. In addition, medications have significant adverse effects with the most common side effects including delayed onset of sleep and decreased appetite both known to potentiate symptoms of ADHD. This results in discontinuation of medication as a result of side effects and the parents’ perception that the medication is ineffective; another factor in medication cessation is the cost burden for families [15,16]. Thus, families are seeking different approaches to treatment, including complementary and alternative therapies (CAM).

The 2007 National Health Interview Survey discovered that approximately 12 percent of children in the United States have used or been given a CAM therapy [17]. More than 50% of Children with ADHD have been reported to use CAM therapies, but only 11% of parents discuss CAM therapies with their child’s physician [18]. As physicians are reluctant to incorporate CAM therapies because they question their scientific efficacy, there is continued need for research and education of these CAM therapies. There is a subset of pediatricians who utilize both conventional and CAM therapies, a field known as integrative medicine, in order to improve outcomes in children with ADHD. Integrative medicine incorporates evidence-based medicine, both conventional and complementary and alternative therapies, to deliver personalized care to the patient, emphasizing diet, nutrients, gut health, and environmental influences as a means to decrease symptoms associated with chronic disorders. Pediatric integrative medicine (PIM) is a fairly new subspecialty and is defined as a relationship-centered practice that utilizes the best evidence in therapeutic approaches to achieve optimal health for children [19]. This review is designed to educate healthcare professionals on a PIM approach to ADHD to help initiate discussions of CAM use with patients and families.

## 2. Diet

What children eat has a profound effect on their health. Although controversial, dietary therapies have been suggested to play a major role in ADHD and should be considered in the evaluation and management of children with ADHD [20,21,22]. Nutritional deficiencies from a “Western” diet, food insecurity, artificial food additives and dyes, and food sensitivities and allergies have been implicated in ADHD [23,24,25,26,27,28]. Most research related to dietary constituents has focused on restricted elimination diets (RED), sugar restriction, and artificial food color exclusion diets in the treatment of ADHD. A research review identified that dietary constituents can significantly worsen ADHD symptoms in 17 of 23 controlled studies [29]. As oxidative stress, or inflammation, is an underlying risk factor in ADHD, modulation of systemic inflammation through nutrition may have potential in decreasing symptoms of youth with ADHD [30]. A Western diet that is high in omega-6 fatty acids, sodium, and sugar intake can induce inflammation, promoting upregulation of pro-inflammatory TH17 cells, cytokines, and IL-10 [31].

### 2.1. Restricted Elimination Diet (RED)

Although adherence is difficult and time consuming, RED has been shown to benefit children with ADHD. The Impact of Nutrition in Children with ADHD (INCA) was a double-blind crossover research study that showed children ages 4–8 years old improve on the abbreviated Conners’ scale by 11.6 after being placed on a tailored RED for five weeks [26]. Furthermore, 63% of the children relapsed with ADHD symptoms after a food challenge. The INCA study found that IgG blood levels did not correlate with ADHD symptoms. This study was well designed with good methodology, large sample size, and correlation with IgG food antigen blood tests. In addition, the diets were specifically tailored to each participant, which helped improve adherence [26]. Due to the recurrence of ADHD symptoms after a five-week RED, more research trials need to investigate how long children with ADHD need to stay on a RED in order to prevent recurrence of symptoms during a food challenge. Some review articles evaluating RED to treat ADHD have discussed that the diet may only influence some aspects of ADHD, such as behavior, and that it should only be done in a select few and for a short period of time of 2–3 weeks [20]. However, antibodies have a half-life between 22–96 days and more improvement in symptoms may be seen with a three-month RED [32]. After approximately 3–6 months, those children who show improvements in behavior and a decrease in ADHD symptoms on RED should introduce one food at a time every one to two weeks until offending foods or food items are identified.

### 2.2. Junk Food Diet

PIM physicians begin with a whole foods, non-processed diet for children with ADHD [33]. Children with ADHD symptoms are at increased risk for becoming obese in adolescence and this is correlated with poor dietary choices and physical inactivity [34]. Children today are exposed to aggressive advertising for highly processed and sugary foods, influencing their eating behaviors [35]. A diet high in processed foods and sugar, otherwise known as “junk food,” has been studied in children with ADHD, though very little evidence has demonstrated associations. One study demonstrated a modest association between children who ate junk food and hyperactivity symptoms, but attenuated after adjustment for confounders [36]. In two separate research studies, sucrose was associated with an increase in motor activity and was shown to reduce attention in children with ADHD, but not in normal children [37,38]. There has been little evidence to support an association between sugar intake and behavioral problems [38,39]. These research studies have not found any association on sugar consumption and ADHD, but cannot rule out a small effect on subsets of children with ADHD [38,39]. In addition, these research studies used artificial sweeteners as the placebo group. This may confound the results because artificial sweeteners have been implicated in neuropsychiatric dysfunction [40,41]. These few research studies also evaluated response to acute effects of sugar consumption, but did not evaluate chronic effects. Further research should evaluate not just sucrose consumption, but excess and chronic carbohydrate consumption and its effect on ADHD symptomology as providers report improvements in behavior with a whole foods diet.

## 3. Gluten-Related Disorders

Gluten-free diets have been implemented for children with neuropsychiatric disorders, including autism and ADHD [7]. Gluten-related disorders, such as celiac disease (CD) and gluten sensitivity, may be associated with ADHD. CD results in gluten-triggered autoimmune intestinal villi destruction in genetically susceptible individuals. Non-celiac gluten sensitivity (NCGS) is a condition in which gluten ingestion leads to morphological or symptomatic manifestations despite the absence of CD. NCGS and CD are triggered by the ingestion of gluten, or more specifically gliadin, the protein components of wheat, rye, and barley. Exposure results in a variable degree of intestinal damage that can induce intestinal permeability, resulting in extra-intestinal manifestations. These extra-intestinal symptoms can masquerade as behavioral and psychiatric disorders.

In the 1950s and 1960s, physicians first began reporting neurologic and psychotic symptoms in patients with CD [42]. There has been emerging evidence to show that individuals with gluten-related disorders, such as CD and NCGS, have neurologic, psychiatric and mood disorders [43,44,45]. Gluten-free diets are becoming widely accepted in the United States [46]. Furthermore, avoiding gluten is common among non-CD children who exhibit nonspecific behavioral and gastrointestinal symptoms [47]. However, there are few research trials done specifically in regards to gluten and its association with ADHD. Dr. Helmut Neiderhofer, MD, PhD and Dr. Klaus Pittschieler, MD demonstrated an increased prevalence of CD in ADHD patients and also noted improved ADHD symptoms after implementation of gluten-free diet in children with CD [48,49]. Inattention has been found to be associated in children with CD compared to healthy children, though a conflicting study by Gungor *et al.* did not show any association between CD and ADHD [48,50,51]. However, it still remains that gluten withdrawal in neuropsychiatric disorders results in improved mood, focus and attention, as well as a decrease in disruptive behaviors [52,53].

The mechanism of action behind gluten’s effect on neuropsychiatric symptoms is still inconclusive, but gastrointestinal immune dysregulation can lead to chronic pro-inflammatory immune dysregulation. This chronic inflammation may be one of the underlying links in the development of ADHD [7,30]. It is also hypothesized that gluten can induce gastrointestinal inflammation, thereby limiting absorption of nutrients and disrupting neurotransmitter metabolism [54].

Despite conflicting evidence of CD and its association with ADHD, clinicians should focus on a broad array of symptoms when evaluating children with ADHD, including both gastrointestinal and neuropsychiatric symptoms to rule out CD or NCGS as an exacerbating condition. Screening for and diagnosing CD and NCGS remains a challenge. A research trial assessed the usefulness of screening for CD when children presented with common CD symptoms (e.g., poor appetite, stomach ache, nausea, bloating, tiredness, hard stools, loose stools and lactose intolerance) [55]. The study results showed that screening children based on only these symptoms did not discriminate between undiagnosed children with CD-associated symptoms and asymptomatic CD children. Determination of NCGS is difficult due to lack of consensus on specific biomarkers, as opposed to CD. Positive effects after gluten withdrawal and return of symptoms with gluten challenge are the main diagnostic criteria [56,57,58]. In most patients with CD and NCGS, the gastrointestinal and extra-intestinal symptoms will reverse on a gluten-free diet [59].

If the decision is made to choose a gluten-free diet, this should be implemented by a team of healthcare professionals, including a trained dietitian, as it is easy to replace wheat/gluten-containing products with gluten-free products high in refined carbohydrates, but low in fiber and nutrients. And if a gluten-free diet is not a good fit, a whole foods diet rich in fresh unprocessed foods is ideal. Most patients with ADHD would benefit from a whole foods diet.

## 4. Micronutrients

The value of nutrition is increasingly becoming recognized as a means for achieving optimal health, as poor diet and nutritional intake are implicated in chronic disorders [60]. Most studies in ADHD and nutrients have focused on polyunsaturated fatty acids and a few minerals, including magnesium, zinc and iron. However, there are many more micronutrients than those that have been predominantly studied. Micronutrients are utilized as cofactors in enzymatic reactions and play a large role in metabolism, neurotransmission, cognitive function, immune function, and detoxification [61,62]. Instead of looking at only a few micronutrients, a comprehensive but focused nutrient panel may be done for those children who have failed conventional treatment approaches or for those families who are exploring other options besides medication. A comprehensive micronutrient panel may include vitamins, minerals, fatty acids, and amino acids, as the quality of our nutrient supply from food has declined [63].

### 4.1. Zinc

Zinc is known to a play a role in neuropsychiatric disorders and is a micronutrient recognized as participating in metabolism relevant to neurotransmitters, hormones, nutrients, and immune function [64,65]. It also contributes to the structure and function of the brain, forming neural pathways affecting neurotransmission [66]. Zinc deficiency can thus have a significant impact on attention, motor activity, cognition, and behavior. Approximately 12%–66% of the world’s population is at risk of zinc deficiency, and is seen especially in children with ADHD who are vulnerable to poor zinc status [67,68]. Although a Cochrane review of 13 randomized trials did not demonstrate a positive effect of zinc supplementation on mental and motor development, there have been several review articles discussing positive improvements in ADHD symptoms with zinc supplementation [69,70,71]. There have been three positive randomized controlled studies on zinc supplementation *versus* placebo in children with ADHD. Bilici *et al.* demonstrated that zinc decreased motor activity and improved impulsivity, but did not increase attention [72]. Akhondzadeh’s *et al.* showed improvement in total ADHD scores with the addition of elemental zinc 13 mg/day to methylphenidate 1 mg/kg/day *versus* placebo and methylphenidate [73]. In a study by Arnold and colleagues, children on zinc supplementation were able to lower their dose of amphetamine, though zinc did not improve inattention or other ADHD symptoms more than placebo [74]. There is encouraging support for zinc supplementation in a subset of children with ADHD, depending on symptoms and if used in combination with stimulants.

### 4.2. Iron

Iron status is commonly evaluated in children with neuropsychiatric disorders likely due to its role as a cofactor in monoaminergic neurotransmitter metabolism [75]. Research has suggested that iron status should be investigated in order to optimize treatment outcomes with stimulant medication [76]. With the prevalence of sleep disturbance in ADHD, Cortese and colleagues found that serum ferritin levels below 45 microg/L may provide evidence to supplement iron in those children with sleep disorder [77]. There are some significant and non-significant associations between serum ferritin levels and ADHD symptoms, but most of these studies focused on serum ferritin levels without other iron indices. As inflammation and oxidative stress have been implicated in the etiopathogenesis of ADHD, serum ferritin is an inflammatory marker when elevated, which may complicate its evaluation. In addition, excess inflammation and low levels of glutathione, the main body’s detoxifier and antioxidant, can result in iron dysregulation [78]. It may be worthwhile then to evaluate a complete blood count with differential and serum ferritin with iron panel to help guide management in children with ADHD, especially when associated with more complex symptoms, including sleep disturbance.

### 4.3. Vitamin B6

Insufficient Vitamin B6, also known as pyridoxine or pyridoxal-5-phosphate, affects metabolism of polyunsaturated fatty acids, hemoglobin synthesis, and neurotransmission [62]. It was later found that pyridoxine is a cofactor in neurotransmitter metabolism including serotonin, glutamate/GABA, and dopamine. Because pyridoxine has a positive effect on children with ADHD, it is recommended to supplement in order to regulate and normalize ADHD behaviors [79]. In fact, a pharmaceutical medication, metadoxine, is an ion-pair salt of pyridoxine that has shown to improve inattentive symptoms, but only studied in the adult population with ADHD [80]. By regulating neurotransmission, vitamin B6 may improve executive function and symptoms of ADHD. Vitamin B6 is generally safe without serious side effects and should be considered in the evaluation of children with ADHD.

### 4.4. Magnesium

Magnesium is the fourth most abundant mineral and is involved as a cofactor in over 300 enzymatic reactions in the body, including fatty acid, glucose and energy metabolism [62]. Some of these metabolic processes play a vital role in neuronal function and neurotransmitter metabolism [81]. Some investigators have proposed magnesium supplementation is advantageous for ADHD symptoms [33,82,83,84]. Magnesium acts as a neuroprotectant from excessive excitatory neurotransmitters, such as glutamate [85]. Several studies have found low erythrocyte or RBC (red blood cell) magnesium in children with ADHD [68,82,84,86]. Although a systematic review of magnesium therapy for treating ADHD did not show a significant improvement in symptoms, most of these studies had methodological limitations, including many that were not double-blind randomized controlled clinical trials, and magnesium was measured inappropriately [87]. Therefore, RBC magnesium, a sensitive biomarker for magnesium deficiency, should be incorporated into future clinical trials and may be important in the work-up for children with ADHD. Appropriate magnesium supplementation may be a useful adjunct for children on stimulant medication.

### 4.5. Polyunsaturated Fatty Acids

Polyunsaturated fatty acid (PUFAs) disturbance has also been implicated in ADHD, as they are necessary for nerve cell membrane fluidity and neuronal function to support neurotransmission. However, the typical American diet contains an imbalance of omega-6 to omega-3 PUFAs. An elevated Omega-6 to Omega-3 ratio is implicated in inflammation [88,89]. Omega-6 fatty acids derived from canola oil, corn oil, soybean oil, and other vegetable fats are inflammatory in nature as they metabolize downstream to arachidonic acid. Arachidonic acid subsequently produces inflammatory prostaglandins and leukotrienes. Gamma-linolenic acid, an n-6 fatty acid, is the exception to the rule which has been shown to be anti-inflammatory in nature [90]. Limiting restaurant and take-out food is also important in balancing PUFAs to reduce inflammatory omega-6 fats because of the prevalence of use and the tendency to damage fats during high heat cooking conditions.

Omega-3 fatty acids, found in flaxseeds, chia seeds, and fatty fish, metabolize downstream to anti-inflammatory prostaglandins and leukotrienes [89,91]. With the increased prevalence of farmed fish, there are less omega-3s produced than are found in their wild counterparts [92]. Dr. Susan Carlson, PhD and her team have found that DHA, an n-3 fatty acid, is essential in neurocognitive development of children. Her research suggests that DHA should be supplemented throughout the lifecycle due to its importance in neuropsychiatric disorders [93].

Practitioners, including dietitians, can help guide families of children with ADHD to increase balanced portions of healthy fats and decrease the inflammatory fats. As research has identified elevated inflammatory markers in children and adolescents with ADHD, maintaining a healthy omega-6 to omega-3 ratio with a diet containing healthy balances of PUFAs, saturated fats, and phospholipids may be a promising integrative medicine approach to treatment of ADHD [94].

However, some reports using placebo-controlled randomized controlled trials (RCTs) evaluating PUFAs efficacy in treating ADHD differ in conclusions [95,96,97,98,99]. Research studies using PUFAs for ADHD that did not reach statistical significance may likely be due to the different combinations, doses, and frequency of PUFA mixes (ALA/LA/EPA/DHA/GLA ratios), in addition to using a placebo with nutritional properties, such as olive oil and vitamin C [97,98,99,100]. The Cochrane review demonstrated that a subgroup of children with ADHD did show improvement with EPA, DHA, and a small amount of GLA [101]. A meta-analysis also demonstrated an effect size of 0.31 for omega-3 fatty acid supplements [95]. When compared to methylphenidate with an effect size of 0.78, Omega-3 fatty acids as a single supplement are approximately 40% as effective as methylphenidate [95,102]. Also, most of these studies did not evaluate phospholipids, such as phosphatidylcholine, essential in neurodevelopment and has been shown to enhance the effect of omega-3 supplements [103].

In clinical practice, evaluation of fatty acids using a comprehensive serum fatty acid panel or RBC fatty acid panel to guide supplementation may be an effective strategy for optimizing treatment outcomes. Fatty acid synthesis and metabolism also involves several other micronutrients as cofactors for the desaturase and elongase enzymes and these may be evaluated by laboratory testing [62]. Individuals vary in need depending on dietary intake and metabolism of fatty acids. PUFAs and associated micronutrient supplementation should be tailored to the individual using guidelines as reports of adverse events exist in the research literature, such as over supplementation of omega-3 fatty acids may increase bleeding time [104]. However, following a healthcare practitioner’s advice on adding PUFAs and monounsaturated fats from fish, flax and chia seeds, nuts, avocado, extra virgin olive oil, and healthy saturated fats from coconut oil is likely advantageous to a child with ADHD.

### 4.6. Carnitine

Carnitine is a fatty acid transporter that transfers long chain fatty acids across mitochondrial membrane for beta-oxidation. Acetyl l-carnitine is an abundant short chain ester of carnitine, which has an effect on brain metabolism and neurotransmission, specifically the cholinergic and dopaminergic pathways [105,106]. Three placebo-controlled trials on carnitine supplementation in participants with ADHD from 2002 to current have been conducted [107,108,109]. However, only one of these small trials showed an improvement after supplementation with carnitine in attention and decrease in aggression in boys with ADHD [108]. Two studies did not demonstrate a significant benefit, but one study found that it did attenuate the side effects of irritability and headaches of methylphenidate [106,108]. However, these two studies did not distinguish between subtypes of ADHD and did not evaluate carnitine levels at baseline and post-treatment. It has to be considered that these children may not have any nutritional need for carnitine supplementation. Carnitine needs further evaluation in future research studies to adequately assess effects in children with ADHD.

### 4.7. Vitamin D

Vitamin D is now implicated in ADHD [110]. Vitamin D is essential for normal brain development and regulates both the innate and adaptive immune system enhancing neuroprotective mechanisms against inflammation [111,112]. A case-control study in Qatar of 1,331 children ages 5–18 years old demonstrated a higher prevalence of vitamin D deficiency in the ADHD children compared to controls [110]. In 2001, a study done by Taylor *et al.* surveyed parents to compare symptoms of their child with ADHD when they were engaging in leisure activities in a windowless room *versus* in an outdoor setting where natural production of vitamin D will occur. The survey results demonstrated that the outdoor setting was more likely to be chosen by parents to reduce inattentive symptoms [113]. Sunlight and vitamin D may be considered a major participant in improving certain symptoms of ADHD and a decrease in outdoor activity with less sunlight exposure may have contributed to the higher prevalence of vitamin D deficiency in children with ADHD. Supplementing children with vitamin D guided by lab results may be a safe and effective strategy to help improve symptoms.

### 4.8. Iodine

For women of childbearing age, iodine insufficiency is approximately 15% in the United States. Pregnant women in the United States are at slight risk of iodine insufficiency especially in the first trimester [114. Iodine deficiency can be measured by a low urine iodine of less than 100 microg/L [62] In women who are planning to become pregnant and are found to be deficient, supplemental iodine may needed to optimize iodine status to ensure healthy cognitive development of the fetus. In addition, psychomotor development and cognitive performance declines in children with iodine insufficiency and iodine status should regularly be evaluated in children with ADHD. A synthesis of the research on iodine deficiency and its relationship to other disease processes other than thyroid disorders has associated iodine deficiency with the occurrence of ADHD [115].

The recommended daily allowance of up to 150 micrograms/day is meant to prevent goiter or thyroid disease [115]. It should be considered that this is not the optimal dose for all individuals who have different metabolic needs and thyroid function. Serial urine iodine measurements should be done until iodine status normalization. More studies are needed to evaluate iodine status and replacement strategies in children with ADHD, as subnormal levels of iodine can have an impact on a child’s ability to focus and concentrate on school tasks.

## 5. Gut Microbiome

Humans have 100 trillion microbes residing in the gastrointestinal tract, some of which may be commensal, while others are pathologic [116]. Consumption of these healthy commensal microbes known as probiotics, have been shown to modulate brain activity [117]. Research has shown that there is a gut microbiome–mind–body continuum in which the enteric microbiota interact with the neuroendocrine system [118]. The gut microbiome essentially has its own nervous system, as it produces neuroendocrine hormones that transmit information across the microbiome [119]. There is increasing evidence that gut bacteria can modulate mood and behavior via the gut–brain axis [120]. Most of these studies have been done in germ-free animals who exhibited changes in mental status and behavior when exposed to pathogenic bacteria, probiotics, or antibiotics. The establishment of a healthy gut microbiome takes place early in development. Infants who were born via cesarean delivery had less microflora in their intestinal tract than those infants born by vaginal delivery [121]. This is consistent with Cesarean birth as a major risk factor of ADHD [122]. Additional evaluation of gut microbiome in ADHD children is therefore warranted.

As part of the practice at University of Kansas Pediatric Integrative Medicine, evaluation of children with neurodevelopment and mood disorders includes stool analysis for intestinal dysbiosis. Medications, such as antibiotics, can disrupt the gut microbiome by reducing diversity and allowing overgrowth of some pathogenic microorganisms [123]. Gut microbiota, such as Candida, can invade the intestine as a pathogen and may lead to deterioration in ADHD symptoms [124]. A Western diet, even in a short-term period, can alter the gut microbiome, again demonstrating the importance of diet and nutrition as a valuable therapy in ADHD [125]. The evidence surrounding the association with healthy gut microbiome and its relationship with psychiatric and behavioral disorders is increasing. This will present new opportunities for interventions with pre- and probiotics, as they are already in common use among families who use integrative therapies.

## 6. Environmental Toxicity

Industry produces over 83,000 chemicals greater than one million pounds per year with little information about the potential effects on neurodevelopment [126,127]. Approximately 1,000 of these chemicals can affect the nervous system, and at least 200 of these are known neurotoxins [128]. Children are highly susceptible to environmental chemicals, especially during preconception and as an infant through breast milk. Exposures to chemicals can alter epigenetic programming, leading to disruptions in neurodevelopment [129]. Recent research studies have now shown that transgenerational changes may even occur when grandparents of the child are exposed to environmental chemicals. Most testing has been done on animals, but these research studies still provide sufficient results to indicate toxic effects in human neurodevelopment [129]. Children also have a larger body surface area with higher respirations leaving them more susceptible to even low-dose chemical exposures through breathing, dermal contact, and food compared to adults. In a cohort of 607 children aged 7–11 years, investigators found an association between low-level prenatal organochlorine exposure via cord blood and ADHD behaviors in childhood [130]. The risk of ADHD-like behaviors in these children increased by 26% to 92%, according to the level of organochlorine exposure [130]. Organochlorines, including polychlorinated biphenyls (PCBs), are environmentally persistent chemicals that cross the placenta and have been shown to alter neurodevelopment [131]. Organophosphates, widely used as pesticides, remain another important avenue for neurodevelopment toxicity in children [132]. Urinary metabolites of organophosphates were obtained from the National Health and Nutrition Examination Survey (NHANES) on 1,139 children. The analyses demonstrated that the children with higher levels of exposure to organophosphates were more likely to have a diagnosis of ADHD [132]. In children, fruits and vegetables may be the most important source of chronic pesticide exposure [133]. This was demonstrated in a study by comparing organic *vs.* conventional diets in children, attesting that organic diets have a protective effect against exposure to organophosphate pesticides [133]. Recommendations to avoid or wash thoroughly the “dirty dozen” conventional produce and eating more of the “Clean 15” may be a beneficial nutritional approach for children with ADHD [134,135].

In addition to obtaining a thorough diet history in patients with ADHD, environmental exposure history needs to be evaluated and addressed. Some families may have a history of using herbicides, insecticides, chemicals including cleaning supplies, and volatile organic compounds, such as air fresheners in their homes. Education is of utmost importance to keep chemical exposure at a minimum. In fact, healthy housing initiatives are gaining popularity among medical communities to help control and prevent chronic disease in children. Advising families to opt for more natural solutions, such as “green” cleaning products that do not contain toxic industrial chemicals and using sticky traps for pests may be healthier alternatives. Limiting plastic products, such as plastic water bottles and microwaveable plastic containers, may also protect against ADHD, as there are positive associations between ADHD and bisphenol A (BPA) and phthalates commonly found in plastics [136].

Of the heavy metals, lead toxicity has been associated with ADHD and other neuropsychiatric disorders. It is well known that lead can cause cognitive impairment and attention dysregulation [137,138]. In a study of 236 children diagnosed with ADHD, low levels of lead exposure demonstrated an increased risk of ADHD [137]. In 2012, the Advisory Committee on Childhood Lead Poisoning Prevention of the Centers for Disease Control and Prevention (CDC) issued updated and stricter guidelines due to long-term effects on neurodevelopment of even low-level lead exposure [138]. Increasing the awareness of healthcare providers, families and the community to environmental chemical and heavy metal exposures is vital to the adjunctive management of these children with ADHD.

## 7. Neurofeedback

One of the most promising treatments in ADHD is neurofeedback, which is an EEG-based form of biofeedback. Neurofeedback is a safe, non-invasive therapy that restores the brain’s ability to function through altering brain wave patterns by operant conditioning [139]. The individual can achieve self-regulation by responding to feedback from real-time audio-visual information. Quantitative EEG studies suggest that children with ADHD exhibit slower brainwave patterns in brain regions, such as the prefrontal cortex, associated with attention and cognitive executive function which is consistent with single photon emission computed tomography (SPECT) and positron emission tomography (PET) studies where decreased metabolism in prefrontal regions is identified [140]. In 1976, Lubar and Shouse were the first to report positive changes in a hyperkinetic child after training the Sensorimotor EEG rhythm (SMR: 12–14 Hz) [141]. Lubar then went on to develop protocols that inhibited theta (slow waves) and rewarded beta (fast waves involved in focus and attention). The FDA announced in July 2013 the approval of diagnosing ADHD by measuring the theta/beta ratio which has been shown to be higher in children with ADHD than normal children [142]. However, excessive theta/beta ratio may only characterize a subgroup of ADHD, approximately 26% of pediatric ADHD patients, compared with 2.5% in healthy controls [143,144,145]. Some studies have concluded that responders to stimulant medications have an initial excess of slow wave (theta) activity. After treatment with stimulant medication, increased beta waves and reduced theta waves result [146]. Stimulant medications may work not only through regulating neurotransmission, but also by affecting brain waves. A quantitative EEG (*i.e.*, brain map), utilized as initial baseline testing of brainwaves, should also be considered in future research studies as an evaluation tool for identifying responders and non-responders to ADHD medication [147]. Other identified EEG phenotypes of ADHD have also been reported including, excess beta, excess alpha, low alpha peak frequency and hypercoherence [143].

In 2004, Slow Cortical Potential (SCP) neurofeedback was found to improve ADHD symptoms [148]. SCP neurofeedback rewards changes in the polarity of the EEG and was initially used to treat epilepsy [149]. SCP neurofeedback enhances regulation of attention through slowly balancing cortical activation and inhibition. Children with ADHD have a significant reduction in the ability to regulate SCPs. In a RCT trial by Gevensleben and his colleagues, 102 children with ADHD participated in neurofeedback and the control group participated in computerized attention skills training. The neurofeedback group completed one block of 18 theta/beta training sessions and one block of 18 SCP training sessions. At a six-month follow-up, reductions in inattention and hyperactivity/impulsivity were about 25%–35% in the neurofeedback group compared to 10%–15% in the control group with a group effect of 4.72 for inattention and 3.45 for hyperactivity/impulsivity [150]. SCP neurofeedback is recognized as an efficacious treatment for ADHD, though future studies still need to identify a sham control and improve efforts to blind participants, parents, teachers, and technicians [151].

A recent RCT research study on 23 children with ADHD carried out 40 sessions of theta/beta training sessions or methylphenidate and compared the two therapies with a six-month follow-up. Although this study had a limitation of small sample size, and 8 out of 12 participants in the neurofeedback group started medication, neurofeedback training was comparable to methylphenidate in reducing ADHD primary symptoms and associated functional impairment. Academic performance significantly improved in the neurofeedback group compared to the group receiving methylphenidate [152].

Despite promising results among research studies and anecdotal evidence, neurofeedback treatment is not yet accepted as a standard therapy for children with ADHD. This is possibly due to limitations of scientific research in neurofeedback accompanied by the difficulty in conducting a well-designed randomized controlled trial. Sham neurofeedback groups are designed as control groups in neurofeedback research. However, attention by neurofeedback technicians may result in positive gains in the control group. Subjective treatment effects can threaten the validity of neurofeedback research and these include participants, parents, and teachers’ expectations of beneficial outcomes, provider training and experience, environmental stressors surrounding the participant, and participants’ engagement during training. In a research update by Loo and Makeig, although the evidence is growing for scientific quality of research in neurofeedback, more research is needed before neurofeedback can be recommended as a first-line, stand-alone treatment modality [143].

In addition, neurofeedback participants must be encouraged to strive toward mastering engagement and regulation [153]. Participants should be rewarded during neurofeedback therapy to further enhance operant conditioning learning. This raises a question about the feasibility of conducting double-blind placebo-controlled trials. Neurofeedback protocols should be guided based on the individual’s baseline qualitative EEG (QEEG), as one child with ADHD may have a different QEEG than another child with ADHD. Hence, protocols used in research may not be applicable to the individual and therefore, neurofeedback, may need to be considered a personalized therapy for improved clinical outcomes in ADHD.

## 8. Future Research

Some of the research related to integrative therapies for children with ADHD has shown mixed results. Of the integrative medicine approaches, the best evidence is for restricted elimination diets, neurofeedback, omega-3 fatty acid supplements, vitamin B6, and zinc. More rigorous and applicable research trials need to be funded in order to identify effective integrative therapies as standard practice. Some researchers suspect the “placebo effect” is responsible for the improvement seen with some integrative therapies. On the other hand, the “placebo effect” can account for perceived improvement from medications and other conventional therapies.

Unfortunately, research is limited in integrative medicine approaches to ADHD. This may be due to funding of and difficulty in designing randomized controlled trials (RCTs), considered the gold standard of evaluating treatment interventions. Evidence-based medicine was initially designed for drugs and procedures, but has been applied to nutrients and other integrative therapies. The high internal validity of RCTs can impede its external validity, meaning that it may not be generalizable to real-life situations, and applicability to the individual may be questionable. Hence, RCTs may not be the best tool to evaluate integrative therapies compared to other types of study designs, such as a pragmatic study design. For example, a single neurofeedback protocol for the general population may not be appropriate for the individual due to differences in brainwaves, genetics, diet, and environmental conditions. Pragmatic trials may be more efficacious in assessing integrative therapeutic approaches in real-life settings, compared to the controlled environments that do not take place outside of the research trial [154]. Evidence-based medicine was initially designed for drugs and procedures, but has been applied to nutrients. To effectively evaluate nutrients, research trials need to be redesigned to account for the difference between drugs and nutrients [155]. The best evidence, however, for an integrative intervention may be from outcome trials.

## 9. Conclusions

With adverse effects of and poor outcomes in long-term safety studies evaluating ADHD medications, more families are turning to integrative modalities to help decrease symptoms and improve quality of life in their children with ADHD. Integrative medicine may provide an alternative approach to treating ADHD, especially for those classified as non-responders. Because a significant percentage of the population with ADHD is classified as non-responsive, integrative medicine approaches to ADHD have gained popularity among families and practitioners. Because research is limited and outcomes show conflicting results, it is difficult for conventional practitioners to advise ADHD patients and their families when questions about integrative therapies arise. Partnering with an experienced pediatrician of integrative medicine may provide an outlet for treatment options and a trusted source for unanswered questions. Since the numbers of pediatricians of integrative medicine are growing, there is an expanding workforce that can provide guidance to conventional practitioners and families and help with design of future clinical trials in ADHD. Healthcare providers need to be aware of integrative modalities in use by families of children with ADHD in order to keep open communication when providing advice on possible benefits and/or limitations based on current research. This will help foster improved communication between families and patients with ADHD and their practitioners.
